# Unique UV-diffused effluent degradation and antimicrobial properties of green synthesized ZnO and MgO nanoparticles using *Ganoderma adspersum*

**DOI:** 10.1038/s41598-026-49225-4

**Published:** 2026-05-16

**Authors:** Kağan Veryer, Yusuf Zalaoglu, Nazmi Sedefoglu, Duygu Godelek, Mehmet Balcilar, Fuat Bozok

**Affiliations:** 1 Department of Biology, Osmaniye Korkut Ata University, 80000 Osmaniye, Türkiye; 2 Department of Electricity and Energy, Osmaniye Korkut Ata University, 80000 Osmaniye, Türkiye; 3 Department of Physics, Osmaniye Korkut Ata University, 80000 Osmaniye, Türkiye; 4 Postgraduate Education Institute, Osmaniye Korkut Ata University, 80000 Osmaniye, Türkiye

**Keywords:** *Ganoderma*, Metal-oxide nanoparticle, Effluent, Dye degradation, Bioactivity, Biotechnology, Environmental sciences, Materials science, Microbiology, Nanoscience and technology

## Abstract

In the present study, zinc oxide (ZnO) and magnesium oxide (MgO) nanoparticles (NPs) were produced by the eco-friendly green synthesis method using the aqueous extract of *Ganoderma adspersum*. ZnO and MgO NPs were characterized using XRD and SEM, which are among the most well-known structural and morphological characterization methods. Besides, the photocatalytic and antibacterial activities of NPs produced were investigated in detail. XRD measurements confirmed that both nanoparticles were produced as intended. Furthermore, it was determined that the ZnO NPs were in the form of nanonails with a homogeneous distribution, while the MgO NPs appeared to be arranged in a specific order and had an irregular planar morphology by means of SEM measurements. As for the photocatalytic performances of green synthesized ZnO and MgO NPs, it was found out that both NPs had high photocatalytic activity. As for the photocatalytic results, ZnO and MgO NPs produced with *G. adspersum* scavenged approximately 98.7% at 140 min and 91.6% at 200 min. Furthermore, it was determined that the synthesized both NPs exhibited significant antibacterial impacts in terms of inhibition zone against six different microorganisms (*A. baumannii* ATCC BA1609, *E. coli* ATCC BAA-2523, *E. faecalis* ATCC 49452, *P. aeruginosa* NCTC 12924, *S. aureus* NCTC 10788, *Y. enterocolitica* ATCC 27729). Antimicrobial activity investigation showed that ZnO and MgO NPs green synthesized by *G. adspersum* revealed the highest antimicrobial activity against *P. aeruginosa* NCTC 12924 with the inhibition zone value of 12.0 and 10.0 mm, respectively).

## Introduction

Nanoparticles, unlike macromolecules, are solid particles smaller than 100 nm that exhibit unique physical and chemical properties^1,2^. These particles are versatile materials that have applications in many industries (electronics, magnetic, optoelectronics, electrical, food preservation, fertilizer, etc.)^3^. Macro-sized metals can be converted into nanoparticles by various physical and chemical methods, but this process is both expensive and harmful in terms of environmental sustainability^4^. When the literature is examined, methods that support the production of eco-friendly, cheaper and more effective nanoparticles called green synthesis have been discovered^1^. The green synthesis of nanoparticles can be evaluated in 3 groups such as intracellular, intercellular and nanoparticle production with the help of phytochemicals^5^. Plant and fungal extracts are used in the nanoparticle production process, produced with the help of phytochemicals. In previous studies, different metal and metal oxide nanoparticles such as zinc oxide, copper oxide, silver, aluminum, iron, selenium and titanium have been successfully produced with the green synthesis method^3,4,6–8^.

Pollution of our environment is one of the most pressing global challenges of this century^9^. One of the fundamental reasons for environmental contamination that needs immediate attention is water pollution caused by the unregulated or unlawful disposal of industrial hazardous organic dyes. Organic dyes used in textiles and allied industries, such as methylene blue (MB), are considered industrial effluents because of their severe toxic effects, potential for cancer, cytotoxicity, and mutational potential^10,11^.

ZnO NPs are frequently used in many industries (medicine, food, cosmetics, agriculture and other industries)^12^. ZnO NPs that have a very low toxicity, serve humanity as effective photocatalytic, sun protection, antimicrobial and even semiconductor materials due to their large surface areas and different properties than those in macroscale^13,14^. Similarly, MgO NPs are valuable oxide nanoparticles with a wide range of applications due to their high surface areas, thermal stability and antimicrobial effect^15^. In addition, they attract great attention in fields such as environment, medicine, energy, and electronics^16^, and play a vital role in the effective reduction of pollutants in water treatment and air purification systems^17^. Thanks to their antimicrobial properties, they are frequently used in infection control and wound healing processes in medical fields, and in the energy sector, their catalytic properties have the potential to be used in energy storage and conversion systems^16^. Extensive research has also shown that excessive nanoparticle accumulation can have negative effects on the environment although they are beneficial^18^. Nanoparticles are still not fully understood substances, and many mechanisms are still unknown^19^. Moreover, the size, chemical composition, surface morphology, and even the charge of nanoparticles allows their properties, such as optoelectronic, magnetic, and toxicity^12,20^.

The aims of this study are to produce ZnO and MgO NPs by the green synthesis method using the extract of *G. adspersum*, to characterize these produced nanoparticles, and to investigate the antimicrobial and photocatalytic activities of the characterized nanoparticles. The present study is novel because it uses *G. adspersum* macrofungi extract, including bioactive compounds, which have never been employed as a reducing agent for zinc acetate and magnesium nitrate to synthesize ZnO and MgO NPs in an environmentally friendly manner. This study is the first report on the photocatalytic performance (on MB dye degradation) under UV illumination using MgO and ZnO NPs synthesized with *G. adspersum* collected from Osmaniye province (The East Mediterranean Region) of Türkiye. Finally, the comprehensive report differs positively from previous work^21–23^ in terms of the photocatalytic activity of green-synthesized metal oxide nanoparticles.

## Materials and methods

### Mushroom sampling and extraction

*Ganoderma adspersum* samples were collected from Kadirli district of Osmaniye province, and deposited at Osmaniye Korkut Ata University Department of Biology, Türkiye, with identification number FBozok00603 (37$\:^\circ\:$21′42.4″ N, 36$\:^\circ\:$07′54.8″ E, 240 elev.). The collected mushroom samples were dried in the laboratory environment in the shade away from the sun. Then, the dried mushrooms were ground into powder with the help of a grinder. 15 g of the powdered samples were taken, 500 mL dH_2_O was added and boiled at 100 °C for 1 h. The samples were then left to cool at room temperature and filtered with Whatman No. 1 filter paper after cooling. The filtered samples were stored in the refrigerator at + 4 °C until used.

### Synthesis and characterization of nanoparticles

50 mL of mushroom extract was taken and 5 g Zn(CH_3_COO)_2_.2H_2_O and Mg(NO_3_)_2_.6H_2_O were added separately. Then, the mixtures obtained were continuously stirred on a hot plate with the help of a magnetic stirrer and kept at 100 °C until the liquid part evaporated. The obtained powders were calcined by burning in a muffle furnace at 800 °C. The synthesized MgO and ZnO NPs were structurally characterized by XRD analysis with a step interval of 0.02° at 2θ angles between 30°–90° and 30°–70°, respectively. Also, the surface morphology of MgO and ZnO NPs was examined using Zeiss Gemini Scanning Electron Microscopy (SEM) at about 50,000× magnification.

### Photocatalytic activity

The photocatalytic performance of ZnO and MgO NPs synthesized via aqueous extract of *G. adspersum* was evaluated through the photodegradation of methylene blue (MB) under ultraviolet (UV) illumination. A stock MB solution (0.008 g/L) was freshly prepared using distilled water. For each trial, 100 mL of the dye solution was transferred into a beaker and mixed with 0.1 g of the respective nanoparticle catalyst^24^. The suspension (dye and nanoparticle) was magnetically stirred in handmade photocatalytic reactor equipped with a 300 W Osram Ultravitalux lamp to ensure homogeneous dispersion and irradiation. Before UV illumination, the mixture was maintained in the dark medium to achieve adsorption–desorption equilibrium. After the light was switched on, 3 mL aliquots were taken at regular intervals (every 20 min) and centrifuged at 10,000 rpm for 5 min to separate the catalyst. The absorbance of the clear supernatant was measured in the range of 400–800 nm using a UV–Vis spectrophotometer (Jasco V-730; blank: distilled water), and the main absorption peak of MB was monitored at 664 nm. This sampling process was continued until the dye solution approached its near-complete decolorization limit for each catalyst type. The photocatalytic degradation efficiency (η) was determined using Eq. (1), based on the change in absorbance of MB at 664 nm over time.

1 $$\:\:\:\eta\:\:\left(\%\right)\:=\:(1\:-\:\frac{C_t}{C_0})\:\times\:\:100$$

where $\:{C}_{0}$ and $\:{C}_{t}$ represent the initial and time-dependent absorbance values, respectively.

### Antibacterial activity

Kirby-Baurer disk diffusion method was used to investigate the antibacterial activity of nanoparticles produced by green synthesis method using *G. adspersum* aqueous extract on six bacterial strains (*Acinetobacter baumannii* ATCC BA1609, *Escherichia coli* ATCC BAA-2523, *Enterococcus faecalis* ATCC 49452, *Pseudomonas aeruginosa* NCTC 12924, *Staphylococcus aureus* NCTC 10788, *Yersinia enterocolitica* ATCC 27729). These bacterial strains were first grown in Luria-Bertani-broth solution until McFarland turbidity was formed and then 1 mL of this solution was spread to cover the entire surface of Mueller-Hinton agar (MHA). Sterile disks were impregnated with NPs (30 µl of a concentration of 50 mg/mL), and the disks were placed on MHA. Then, they were kept in the incubator for 24 h. The inhibition zones formed were measured using a ruler.

### Statistical analysis

Within the scope of this study, the SPSS software package (Version 20.0, IBM Statistics, USA) was used to evaluate the antimicrobial activities of Ag nanoparticles obtained by green synthesis methods using different plant extracts. A one-way analysis of variance was performed on the results obtained, and Duncan’s test was applied at a 95% confidence interval.

## Results and discussion

### XRD analysis

**Fig. 1:**
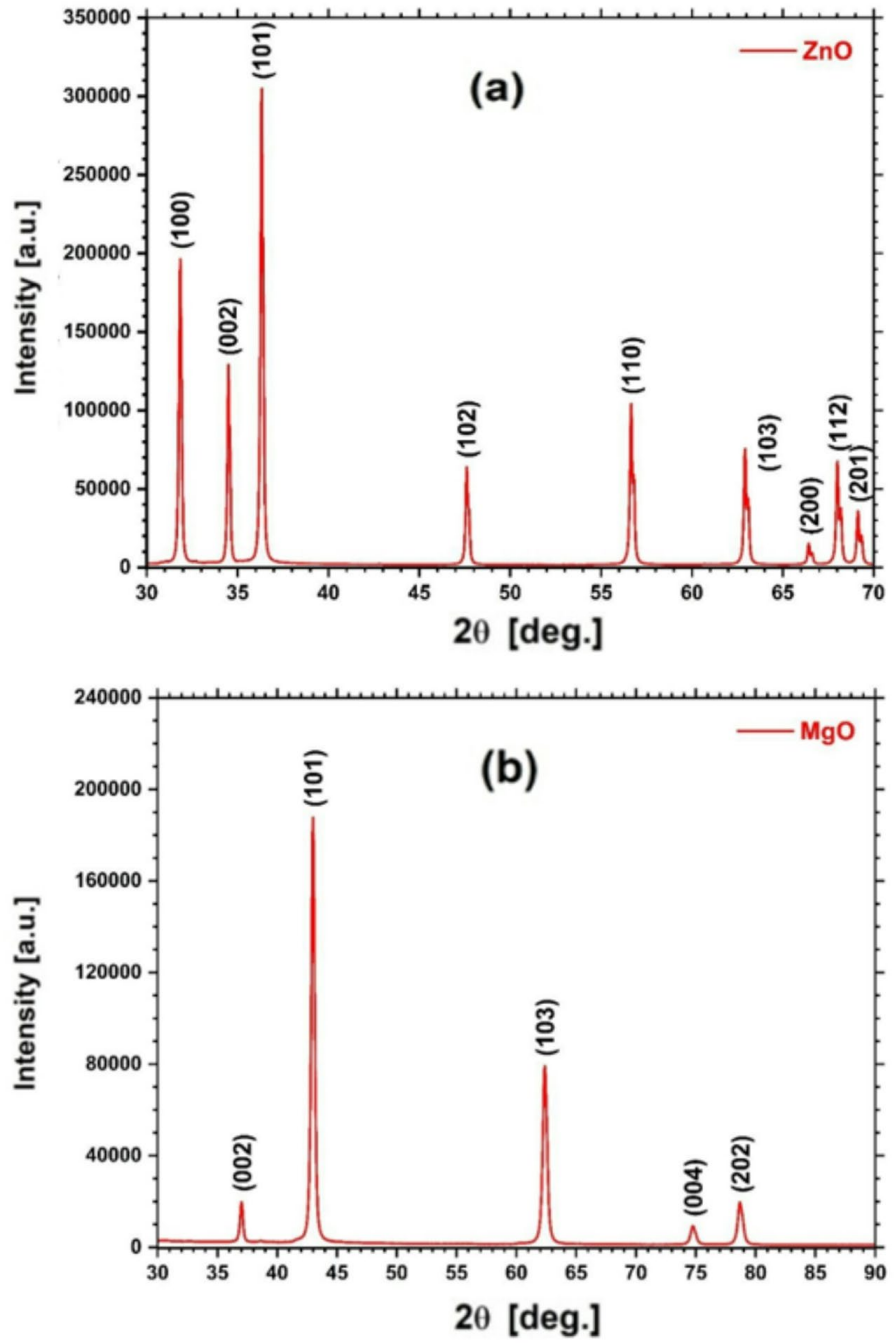
X-ray diffraction patterns of the (**a**) ZnO and (**b**) MgO NPs via green synthesis method collaborating *G. adspersum* extract.

XRD measurements of MgO and ZnO NPs obtained via green synthesis technique by means of *G. adspersum* extract are given as Fig. 1 at 2θ angle values between 30°-90° and 30°-70° with a step interval of 0.02°, respectively. When the corresponding values in the JCPDS database of the diffraction peaks observed in the XRD graphs of the samples were examined, it was found that they were compatible with JCPDS card No. 75–0447^25,26^ for MgO nanoparticle with cubic crystal structure and JCPDS card No. 36-1451 for ZnO nanoparticle with hexagonal wurtzite structure^27,28^. As for the 2θ angle values of the peaks in the X-ray diffraction curves, these angle values for **[[MgO]]**//*[[ZnO]]* NPs were numerically determined as **[[37.02°**,** 43.01°**,** 62.39°**,** 74.75°**,** 78.70]]**//*[[34.85°*,* 34.50°*,* 36.34°*,* 47.60°*,* 56.66°*,* 62.94°*,* 66.43°*,* 68.01°*,* 69.14°]]*. Besides, in accordance with the 2θ angle values given above, **[[MgO]]**//*[[ZnO]]* NPs were observed to be in the **[[(002)**,** (101)**,** (103)**,** (004)**,** (202)]]**//*[[(100)*,* (002)*,* (101)*,* (102)*,* (110)*,* (103)*,* (200)*,* (112)*,* (201)]]* diffraction planes, respectively (Fig. 1a,b). Furthermore, we can absolutely say that MgO and ZnO NPs were obtained completely from the diffraction curves observed in Fig. 1a,b. In addition, the narrow and high-intensity peaks for MgO and ZnO NPs obtained using *G. adspersum* extract in the diffraction patterns shown in Fig. 1a,b clearly indicate that both nanoparticles have high crystallinity. Furthermore, the absence of peaks other than those given in the card numbers mentioned above for the MgO and ZnO NPs produced confirms that our samples have a high-purity crystal structure. The average particle sizes of MgO and ZnO NPs were calculated using the Debye-Scherrer equation (Eq. 2) given below^29,30^:

2 $$\:D=\frac{k\lambda\:}{\beta\:\mathrm{cos}\theta\:}$$

In the above Eq. (2), D represents the calculated average grain size of the NPs, while k represents the Scherrer constant and is acquired as 0.9 numerically. Also, λ is the wavelength of the applied X-ray, β is the value of the peak width at half point of XRD peak in radians (FWHM), and θ is the Bragg diffraction angle. As for the calculation of average grain size of the nanoparticles, these values are numerically given as 23.36 nm and 48.95 nm, respectively. Moreover, lattice parameters of MgO (Eq. 3) and ZnO (Eq. 4) NPs were calculated with the equation given below^28,31^.

3 $$\:\frac{1}{{d}^{2}}=\left(\frac{{h}^{2}+{k}^{2}+{l}^{2}}{{a}^{2}}\right)$$

4 $$\:\frac{1}{{d}^{2}}=\frac{4}{3}\left(\frac{{h}^{2}+{k}^{2}+hk}{{a}^{2}}\right)+\frac{{l}^{2}}{{c}^{2}}$$

The average lattice parameter of the MgO nanoparticle (which has a cubic structure, so a = b=c) was found to be 4.21 Å. The lattice parameter of the ZnO nanoparticle was calculated as 5.195 Å for the “*c*” lattice parameter for the 002 peak, while “*a*” lattice parameter was calculated as an average of 3.245 Å for the (100), (110), and (200) planes.

Shortly, it is clearly seen that the average particle size of ZnO NPs is more than twice MgO NPs. This situation shows that the *G. adspersum* extract has different effects on the production of different metal oxide NPs.

### SEM analysis

**Fig. 2:**
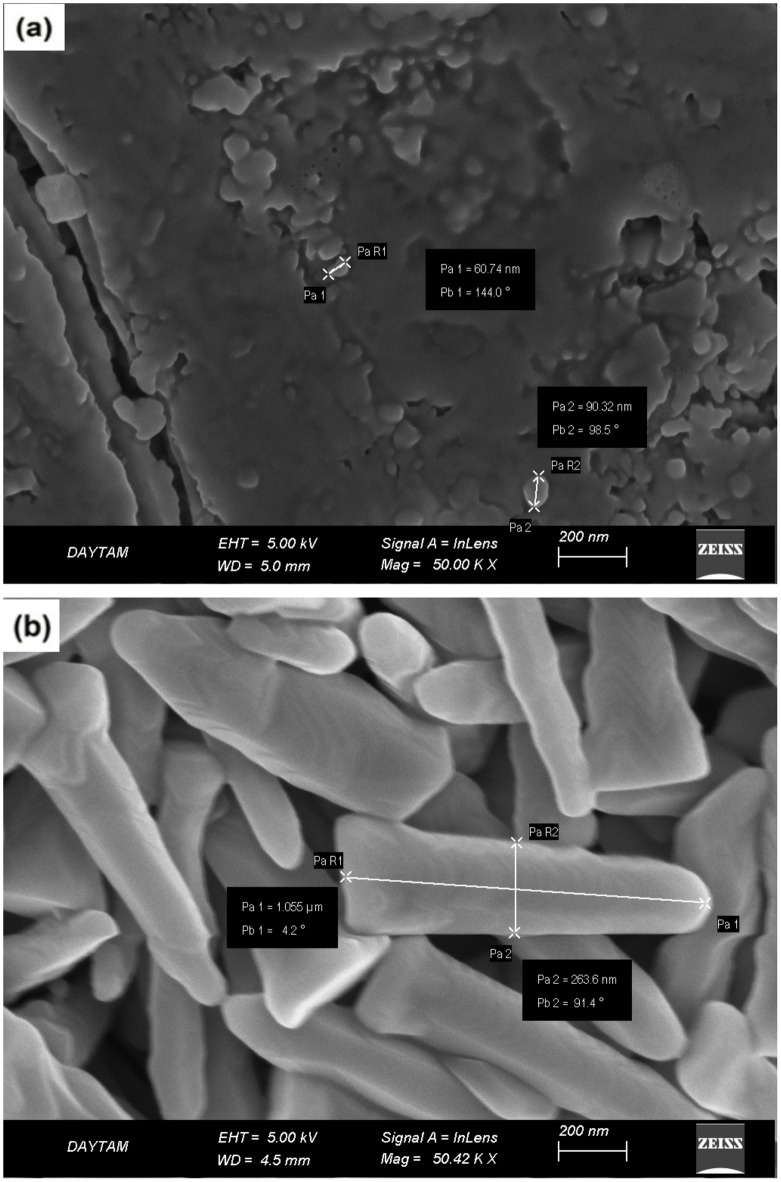
SEM images of the (**a**) MgO and (**b**) ZnO NPs by means of eco-friendly green synthesis technique accompanied with *G. adspersum* extract.

The surface morphology of MgO and ZnO NPs obtained by means of the green synthesis technique with *G. adspersum* fungal extract was examined using Scanning Electron Microscopy (SEM). The pictures of the MgO and ZnO NPs were achieved in Zeiss Gemini FESEM scanning electron microscope at 50000x magnification. Also, the images obtained from the surfaces of the MgO and ZnO NPs are shown in Fig. 2a,b, respectively. As can be seen in Fig. 2a, the MgO NPs appear to be arranged in a specific order and have an irregular planar morphology. Furthermore, it was determined that a single large nanoparticle forms on a single plane due to the aggregation of smaller nanoparticles, almost adherent. In contrast, the SEM images of the ZnO NPs revealed a homogeneous distribution of nanoparticles, and their shapes were all nanonail-like nanoparticles (Fig. 2b). In addition, the nanonail-like ZnO NPs were observed to be composed of two parts: (I) a nanowire at the bottom of the body, and (II) an ultrathin symmetric circular cap at the top of the tip^32–35^. Although the ZnO NPs, shaped nanonail-like, appear to be arranged in various ways in the photographs, they are ordered so tightly together that there is virtually no space between them. Therefore, based on the images of the ZnO nanonails we produced, it would not be wrong to expect that analyses conducted regarding the technological and industrial uses of ZnO nanonails will yield positive results. As the particle sizes of the produced NPs were compared, it is revealed that the particle size of the MgO NPs is smaller than that of the ZnO NPs due to agglomeration. This situation confirms the grain size values calculated using XRD analysis.

### Photocatalytic activity

The photocatalytic activities of the ZnO and MgO NPs produced within the scope of this study were investigated by examining their effect on the MB dye, which is frequently used in industrial applications, under UV light at room temperature. In this analysis, we sought to determine in detail how effective the nanoparticles we produced could be in removing the MB dye, which is used as an organic pollutant, from the solution prepared. First, the nanoparticles and the solution prepared with MB were kept in a dark environment to ensure that the results of the absorbance measurements we would perform with the UV–Vis spectrophotometer were accurate and that equilibrium could be achieved in the measurements. Subsequently, the prepared solution was removed from the dark environment and exposed to UV light in a closed box to enable the photocatalytic mechanism. According to this mechanism, when the photon energy from the UV lamp is equal or greater than the excitation energy in the nanoparticles, the electrons in the valence band of the nanoparticles move to the conduction band, electron/hole $\:\left({e}^{-}/{h}^{+}\right)$ pairs are produced as given in Eq. (5). Then, electrons $\:\left({e}^{-}\right)$ react with dissolved oxygen to produce superoxide radical anions $\:\left({{O}_{2}\cdot\:}^{-}\right)$, while $\:{h}^{+}$ reacts with water and hydroxide ions to form hydroxyl radicals $\:\left(OH\cdot\:\right)$. Furthermore, the superoxide radicals that appear in Eq. (6) react with $\:{H}^{+}$ to produce hydroperoxyl radicals $\:\left(H{O}_{2}\cdot\:\right)$. Then, two hydroperoxyl radicals combine through a chemical reaction to form hydrogen peroxide $\:\left({H}_{2}{O}_{2}\right)$. The hydrogen peroxide and superoxide radicals present in the medium react to form hydroxyl radicals $\:\left(OH\cdot\:\right)$, which are powerful oxidizing agents (Eq. 10). Moreover, hydroxyl radicals are produced by means of the reaction between hydrogen peroxide and UV light. Therefore, the resulting hydroxyl radicals having high oxidative potential will remove organic pollutants attached to the surface of nanoparticles, and this removal will result in green compounds such as $\:C{O}_{2},\:{H}_{2}O$, etc. Thus, the mechanism of the process occurring for the photocatalytic activities of organic compounds via redox reaction under a high-energy light source can be summarized by the Eqs. (5)–(12) given below^24,36^:

5 $$\:ZnO+h\nu\:\:\left(UV\:light\right)\to\:{e}^{-}+{h}^{+}$$

6 $$\:{e}^{-}+{O}_{2}\to\:{{O}_{2}\cdot\:}^{-}$$

7 $$\:{h}^{+}+{H}_{2}O+O{H}^{-}\to\:2\left(OH\cdot\:\right)+{H}^{+}$$

8 $$\:{{O}_{2}\cdot\:}^{-}+{H}^{+}\to\:H{O}_{2}\cdot\:$$

9 $$\:H{O}_{2}\cdot\:+H{O}_{2}\cdot\:\to\:{H}_{2}{O}_{2}+{O}_{2}$$

10 $$\:{H}_{2}{O}_{2}+{{O}_{2}\cdot\:}^{-}\to\:OH\cdot\:+O{H}^{-}+{O}_{2}$$

11 $$\:{H}_{2}{O}_{2}+h\nu\:\:\left(UV\:light\right)\to\:2\left(OH\cdot\:\right)$$

12 $$\:Organic\:pullutants\:\left(MB\right)+OH\cdot\:\to\:{CO}_{2}+{H}_{2}O$$

**Fig. 3:**
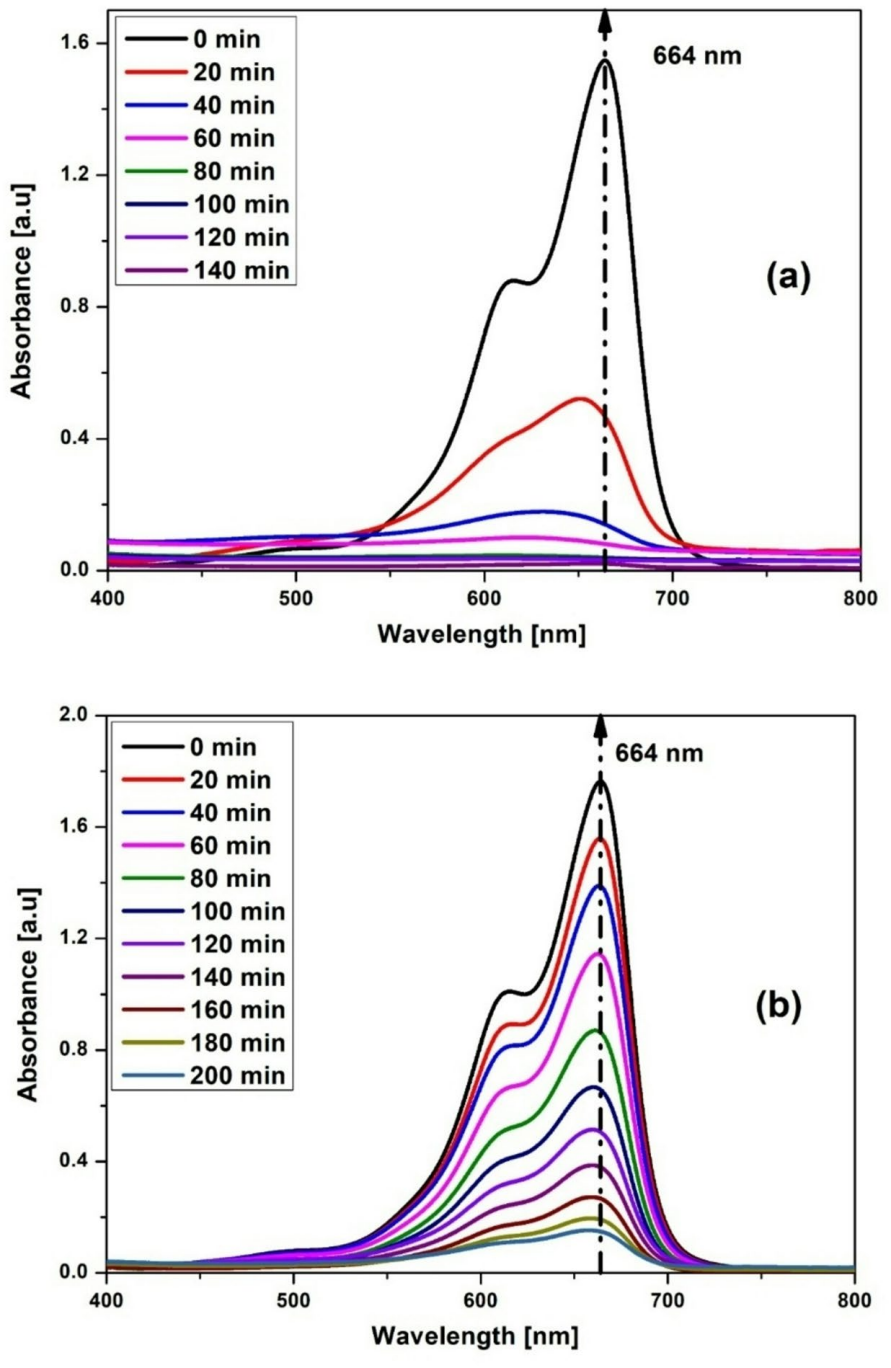
Absorbance vs. wavelength graphics of MB after UV irradiation (**a**) ZnO and (**b**) MgO NPs.

Furthermore, MB was used to evaluate the photocatalytic activities of ZnO and MgO NPs by monitoring the degradation of dye under visible light irradiation at different time intervals. The UV–Vis absorption spectra of the reaction mixtures are presented in Fig. 3a,b. Both catalysts exhibited a characteristic absorption peak at approximately 664 nm, corresponding to the λ_max_ of MB. As the irradiation time increased, the absorbance intensity at 664 nm gradually decreased, indicating the progressive degradation of the dye molecules due to photocatalytic activity. In the case of ZnO NPs (Fig. 3a), a sharp decrease in absorbance was observed within the first 60 min, followed by a slower rate of degradation, which plateaued around 140 min. Conversely, MgO NPs (Fig. 3b) demonstrated a more gradual but continuous decrease in absorbance up to 200 min, maintaining catalytic activity over a longer period. Photocatalytic activity of ZnO and MgO NPs was calculated as approximately 98.7% at 140 min and 91.6% at 200 min, respectively. It should also be noted that while ZnO NPs purified the wastewater by approximately 69.8% during the first 20-minute cycle, the purification performance of MgO NPs remained at 11.6% in the first cycle. Further, both MgO and ZnO NPs effectively facilitated the degradation of MB dye, confirming their potential as photocatalytic agents. When previous studies reported in the literature are examined, it is suggested that ZnO exhibits faster photocatalytic activity due to its high electron–hole separation efficiency^35,37,38^. The findings of the present study similarly demonstrate a rapid initial photocatalytic response by ZnO, thereby supporting the existing literature. In contrast, MgO exhibits a slower dye degradation rate because of its wide band gap, high surface area stability, alkalinity and rich oxygen vacancies^39^. As a result, it is thought that homogenous nanonail-like ZnO NPs produced by the green synthesis method exhibit faster photocatalytic activity than MgO NPs due to their high surface area^40,41^.

### Antimicrobial activity

In the presented study, ZnO and MgO NPs were produced by green synthesis technique using the aqueous extract of *G. adspersum* mushroom and their antimicrobial activities on various microorganisms (*Acinetobacter baumannii* ATCC BA1609, *Escherichia coli* ATCC BAA-2523, *Enterococcus faecalis* ATCC 49452, *Pseudomonas aeruginosa* NCTC 12924, *Staphylococcus aureus* NCTC 10788, *Yersinia enterocolitica* ATCC 27729) were investigated (Table 1). When the table is examined carefully, it can be said that both ZnO and MgO NPs produced by green synthesis technique using *G. adspersum* aqueous extract have moderately antimicrobial activity on the tested strains. The produced MgO NPs created the highest inhibition zone (12.0 mm) in *Pseudomonas aeruginosa* NCTC 12,924, followed by *Acinetobacter baumannii* ATCC BA1609 (11.0 mm) > *Enterococcus faecalis* ATCC 49,452 (10.0 mm) > *Escherichia coli* ATCC BAA-2523 (8.0 mm) = *Staphylococcus aureus* NCTC 10,788 (8.0 mm) > *Yersinia enterocolitica* ATCC 27,729 (7.0 mm), respectively. ZnO NPs created the highest inhibition zone in *Enterococcus faecalis* ATCC 49,452 (10.0 mm) = *Pseudomonasa eruginosa* NCTC 12,924 (10.0 mm), followed by *Acinetobacter baumannii* ATCC BA1609 (9.0 mm) > *Escherichia coli* ATCC BAA-2523 (8.0 mm) = *Staphylococcus aureus* NCTC 10,788 (8.0 mm) > *Yersinia enterocolitica* ATCC 27,729 (7.0 mm), respectively. In previous studies, the antimicrobial activities of different nanoparticles produced by green synthesis technique using various fungi were determined^42–46^. In addition, ZnO and MgO NPs were synthesized using different organisms and their various activities were investigated^47–51^. In the study conducted by El-Sayyad et al.^52^, MgO was synthesized using the extracted melanin pigment of *Penicillium chrysogenum* provided by the Pharmaceutical Microbiology Laboratory (NCRRT, Cairo, Egypt) and the characterization of this synthesized nanoparticle was carried out by UV–Vis, DLS, XRD, FTIR and TEM analyses. The antimicrobial activity of the characterized MgO NPs was investigated on *Escherichia coli*,* Enterobacter cloacae*,* Acinetobacter baumannii*,* Enterococcus faecalis*,* Staphylococcus aureus* and *Staphylococcus epidermidis* microorganisms. Consequently, it was emphasized that MgO NPs could be promising antimicrobial agents against *Enterococcus faecalis*, *Candida albicans* and *Klebsiella pneumoniae*. According to Vasanth Kumar et al.^53^, the extracellular products of the endophytic fungus (98.9% *Aspergillus* sp. were determined as a result of molecular characterization) isolated from the brown algae *Dictyota dichotoma* collected from the Gulf of Mannar coast of Rameswaram district, Tamil Nadu, India were extracted and ZnO NPs were synthesized using this extract. The synthesized ZnO NPs were characterized by UV–Vis, FTIR, FESEM-EDX analyzes and their antibacterial, antibiofilm and photocatalytic activities were investigated. Finally, it was determined that the synthesized nanoparticles showed significant antibacterial effects in terms of inhibition zone and antibiofilm activity against pathogenic bacteria.

**Table 1:** Antibacterial activity values of MgO and ZnO NPs against various bacterial strains.

Species	Inhibition zones (mm)	
	MgO NPs (30 µL*)	ZnO NPs (30 µL*)
*Acinetobacter baumannii ATCC BA1609*	11.0 ± 1.0^b^	9.0 ± 2.0^ab^
*Escherichia coli ATCC BAA-2523*	8.0 ± 0.5^bc^	8.0 ± 0.5^ab^
*Enterococcus faecalis ATCC 49,452*	10.0 ± 2.0^ab^	10.0 ± 1.0^a^
*Pseudomonas aeruginosa NCTC 12,924*	12.0 ± 1.0^a^	10.0 ± 1.0^a^
*Staphylococcus aureus NCTC 10,788*	8.0 ± 1.0^bc^	8.0 ± 1.0^ab^
*Yersinia enterocolitica ATCC 27,729*	7.0 ± 1.0^c^	7.0 ± 1.0^b^

*Stock concentration: 50 mg/mL.

## Conclusion

Within the scope of this study, ZnO and MgO NPs were produced by the green synthesis technique using the aqueous extract of *G. adspersum* mushroom. The characterization of both green-synthesized nanoparticles was carried out by XRD and SEM analysis. Also, the photocatalytic and antimicrobial performances of both NPs were investigated, and the results can be given as follows:

- The structural analysis of MgO and ZnO NPs produced by green synthesis technique with *G. adspersum* mushroom extract was performed by X-ray diffraction. Briefly reviewing the XRD results, it was seen that the crystal structure of both nanoparticles was fully obtained with a high purity crystal structure. As the calculation of the crystal sizes of both nanoparticles via X-ray diffraction peaks, it was seen that the crystal size of the ZnO NPs was greater than the crystal size of the MgO NPs.
- Besides, in the morphological analysis performed by SEM analysis, it was observed that the particles of the MgO NPs had an irregular planar morphology in a certain order and that smaller particles were almost adherent and agglomerated. On the other hand, in the morphological analysis of the ZnO NPs, it was observed that the distribution of the nanoparticles was homogeneous, and all particle shapes were in the form of nanonails.
- Dye degradation performance of green synthesized ZnO and MgO NPs were researched, and it was revealed that both NPs exhibited high photocatalytic performance. Further, ZnO NPs had higher and faster (approximately 98.7% degradation in 140 min) MB dye degradation activity than MgO NPs.
- It was determined that both ZnO and MgO NPs synthesized using *G. adspersum* aqueous extract had antimicrobial activity on the tested six microorganisms. As for the analysis results, it was concluded that both ZnO and MgO NPs had moderately antimicrobial activity on all microorganisms used. At the same time, it is recommended that in future studies, bioactivities on various microorganisms should be studied by using the extracts of more than one mushroom rather than a single mushroom, both by synergistic effect and by synthesizing different metal oxides.

## Data Availability

Raw data can be obtained from the responsible author upon request.
